# Genetic and Chemical-Genetic Interactions Map Biogenesis and Permeability Determinants of the Outer Membrane of Escherichia coli

**DOI:** 10.1128/mBio.00161-20

**Published:** 2020-03-10

**Authors:** Kristina Klobucar, Shawn French, Jean-Philippe Côté, James R. Howes, Eric D. Brown

**Affiliations:** a Department of Biochemistry and Biomedical Sciences, McMaster University, Hamilton, Ontario, Canada; b Michael G. DeGroote Institute for Infectious Disease Research, McMaster University, Hamilton, Ontario, Canada; Georgia Institute of Technology School of Biological Sciences

**Keywords:** enterobacterial common antigen, genetic interaction, knockout, lipopolysaccharide, outer membrane, permeability

## Abstract

Gram-negative bacteria are intrinsically resistant to many antibiotics due to their outer membrane barrier. Although the outer membrane has been studied for decades, there is much to uncover about the biology and permeability of this complex structure. Investigating synthetic genetic interactions can reveal a great deal of information about genetic function and pathway interconnectivity. Here, we performed synthetic genetic arrays (SGAs) in Escherichia coli by crossing a subset of gene deletion strains implicated in outer membrane permeability with nonessential gene and small RNA (sRNA) deletion collections. Some 155,400 double-deletion strains were grown on rich microbiological medium with and without subinhibitory concentrations of two antibiotics excluded by the outer membrane, vancomycin and rifampin, to probe both genetic interactions and permeability. The genetic interactions of interest were synthetic sick or lethal (SSL) gene deletions that were detrimental to the cell in combination but had a negligible impact on viability individually. On average, there were ∼30, ∼36, and ∼40 SSL interactions per gene under no-drug, rifampin, and vancomycin conditions, respectively; however, many of these involved frequent interactors. Our data sets have been compiled into an interactive database called the Outer Membrane Interaction (OMI) Explorer, where genetic interactions can be searched, visualized across the genome, compared between conditions, and enriched for gene ontology (GO) terms. A set of SSL interactions revealed connectivity and permeability links between enterobacterial common antigen (ECA) and lipopolysaccharide (LPS) of the outer membrane. This data set provides a novel platform to generate hypotheses about outer membrane biology and permeability.

## Abstract (precis)

Gram-negative bacteria are a major concern for public health, particularly due to the rise of antibiotic resistance. It is important to understand the biology and permeability of the outer membrane of these bacteria in order to increase the efficacy of antibiotics that have difficulty penetrating this structure. Here, we studied the genetic interactions of a subset of outer membrane-related gene deletions in the model Gram-negative bacterium E. coli. We systematically combined these mutants with 3,985 nonessential gene and small RNA deletion mutations in the genome. We examined the viability of these double-deletion strains and probed their permeability characteristics using two antibiotics that have difficulty crossing the outer membrane barrier. An understanding of the genetic basis for outer membrane integrity can assist in the development of new antibiotics with favorable permeability properties and the discovery of compounds capable of increasing outer membrane permeability to enhance the activity of existing antibiotics.

## INTRODUCTION

The Gram-negative outer membrane confers intrinsic resistance to some antibiotics (1, 2). This outer membrane structure is an asymmetric bilayer with a phospholipid inner leaflet and a lipopolysaccharide (LPS) outer leaflet, which is typically composed of lipid A, inner and outer core oligosaccharides, and O-antigenic polysaccharide, the latter of which is absent in K-12 strains of Escherichia coli (3–5). Negatively charged LPS molecules exhibit strong lateral interactions and are stabilized by divalent cations such as Mg^2+^ and Ca^2+^, creating a strong permeability barrier to protect the cell from toxins (5–8). As such, diffusion of hydrophobic molecules through the outer membrane is hindered, and hydrophilic molecules over ∼600 Da are excluded from entry through outer membrane porins (9–11). Thus, any molecules that are hydrophobic or large and hydrophilic are prevented from cell entry, including many antibiotics that are otherwise effective against Gram-positive bacteria, rendering these drugs useless in the treatment of Gram-negative infections (12).

In order to potentiate antibiotics conventionally used to treat Gram-positive infections in Gram-negative bacteria, the permeability of the outer membrane must be altered. As divalent cations that reduce the negative charge of LPS are required for outer membrane stability, chelation of these ions with compounds such as EDTA results in membrane permeabilization (8, 13). Cationic compounds such as polymyxins are also known to bind LPS to physically disrupt the outer membrane (8, 14). Mutations in certain outer membrane biosynthetic genes, such as the LPS inner core, are likewise known to potentiate hydrophobic antibiotics, as these mutants tend to have more phospholipids in the outer leaflet of their outer membranes (5). Additionally, cold temperatures have been shown to increase the rigidity of the Gram-negative outer membrane and increase its susceptibility to “cracking” that compromises the permeability barrier (5). Indeed, we previously demonstrated that E. coli could be sensitized to the Gram-positive-targeting antibiotic vancomycin at cold temperatures (15). In a systematic search for suppressors of this phenotype, we found that deletion of certain outer membrane-related genes leads to antagonism of vancomycin activity in the cold. Since many of these genes encoded LPS biosynthetic functions, we reasoned that altered LPS packing could make the outer membrane more resistant to cracking (15). Although the Gram-negative outer membrane has been studied for decades, there is still much to learn about this complex barrier.

A great deal of information can be uncovered about genes of interest by investigating their synthetic interactions. A synthetic interaction is a phenotype produced by a combination of genetic and/or chemical perturbations that differs from the phenotype expected based on the effect of each perturbation alone (16). Synthetic interactions in which the result is better growth than expected are referred to as suppressing or synthetic viable. Conversely, those interactions that result in worse growth than expected or lethality to the cell are referred to as enhancing or synthetic sick/lethal (SSL) interactions (17–21). SSL interactions tend to occur if both perturbations target genes in parallel or redundant pathways to abrogate a process that is essential for growth (22). Studying these interactions in high throughput using synthetic genetic arrays (SGAs) (17–19) can provide a wealth of complex information about genetic involvement in cellular pathways and cross talk between pathways, which can help characterize genes of unknown function. Furthermore, including small RNA (sRNA) deletions (23) in SGAs can provide some clarity on the regulation of SSL interactions. Previous SGA studies in E. coli have characterized the roles of certain outer membrane proteins (19, 24), iron-sulfur cluster biosynthetic genes (18), ribosome biogenesis factors (25, 26), nutrient stress genes (21), and cell shape-related genes (27). Interactions have also previously been probed under different conditions such as DNA damage (28) and nutrient and temperature stressors (29).

Here, we performed SGAs by focusing on a subset of E. coli gene deletion strains that have been implicated in outer membrane permeability; mutations in these genes led to antagonism of vancomycin activity in cold temperatures (15). We systematically constructed double-deletion strains with these 39 query genes and each of the E. coli single-gene deletion (Keio) (30) and sRNA deletion (23) collections. Other studies have performed genome-wide SGAs by crossing query deletion strains with the Keio collection; however, this study also includes a large collection of sRNA deletion strains, increasing the scope of interactions probed. Once generated, the double-deletion strains were grown on rich microbiological medium with and without subinhibitory concentrations of two Gram-positive-targeting antibiotics, rifampin and vancomycin, to probe both genetic interactions and outer membrane permeability. High-density arrays of double-deletion strains were carefully analyzed for growth (21, 27, 31), and the resulting data set has been compiled into a searchable, interactive database called the Outer Membrane Interaction (OMI) Explorer (https://edbrownlab.shinyapps.io/omi_explorer/), where genetic interactions of these outer membrane-implicated genes can be visualized across the genome. Here, we have also explored a curious synthetic sick interaction in solid medium between strains with a truncated LPS inner core and a deletion of *yhdP*, an enigmatic gene implicated in the stationary-phase stress response (32) and the production of enterobacterial common antigen (ECA) (33).

## RESULTS

### Synthetic genetic arrays of outer membrane-related genes.

In order to gain insight into the permeability of the outer membrane and the interconnectivity of its biosynthetic pathways, we crossed 39 query gene deletion strains, previously implicated in outer membrane permeability in E. coli (15), with genome-wide single-gene and sRNA deletion collections (23, 30). The 39 query gene deletion strains include those involved in LPS biosynthesis, maintenance of lipid asymmetry, ECA biosynthesis, flagellum biosynthesis, curli biosynthesis, efflux pump components, and genes of unknown function (see Table S1 in the supplemental material). These crosses were performed in high throughput using standard SGA procedures (18, 19, 21), whereby a query gene deletion strain was transferred to each gene and sRNA deletion strain using conjugation at a 1,536-colony density to generate double-deletion strains (see Materials and Methods and Fig. S1A and B for the workflow). Double-deletion strains were pinned in quadruplicate to a 6,144-colony density on assay plates containing no drug, 1/8 MIC of vancomycin, or 1/8 MIC of rifampin (see Table S1 for solid-medium MIC values of query strains), and growth was measured at endpoint. Vancomycin and rifampin were chosen as they are both large-scaffold antibiotics with widely different physical-chemical properties that are precluded from entry into Gram-negative bacteria by the outer membrane (34, 35).

10.1128/mBio.00161-20.1 FIG S1 Workflow used to generate SGAs. (A) Steps to perform a single replicate of a query deletion strain crossed with one plate of the Keio collection using the Singer Rotor HDA. Drug plates pinned at a 6,144-colony density are at 1/8 MIC of the query strain. (B) Data analysis steps for determination of synthetic interactions. apra, apramycin; kan, kanamycin; vanco, vancomycin; rif, rifampin. Download FIG S1, TIF file, 2.2 MB. Copyright © 2020 Klobucar et al. 2020 Klobucar et al. This content is distributed under the terms of the Creative Commons Attribution 4.0 International license.

10.1128/mBio.00161-20.7 TABLE S1 Background information on query gene deletion strains. Selected CIP strains used to make query gene deletion strains for Hfr are shown. Primers for amplifying the apramycin resistance cassette to generate deletion strains and confirmation of the cassette are listed. Solid MICs of vancomycin and rifampin are also listed. Download Table S1, XLSX file, 0.02 MB. Copyright © 2020 Klobucar et al. 2020 Klobucar et al. This content is distributed under the terms of the Creative Commons Attribution 4.0 International license.

**FIG 1:**
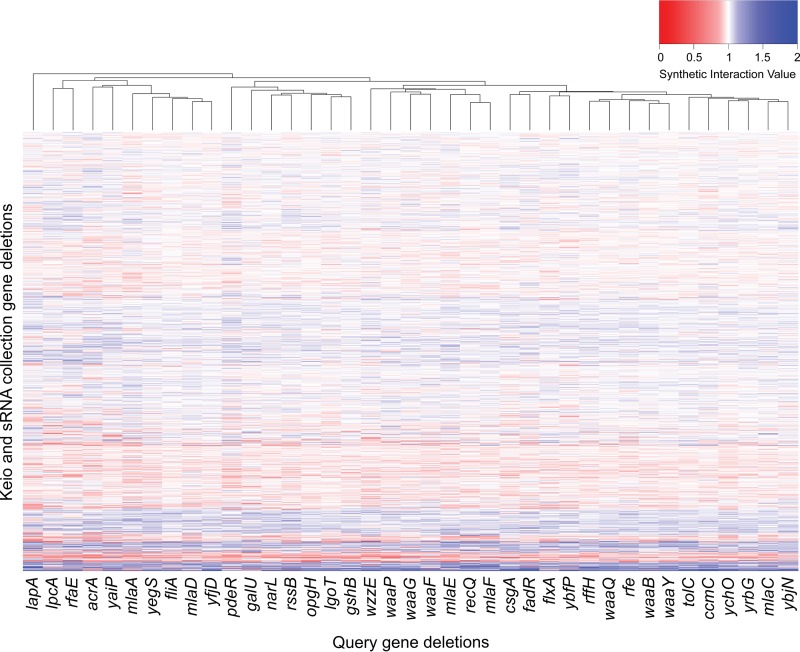
Synthetic genetic arrays of outer membrane-related gene deletions. A heat map shows the genome-wide SIVs for each double-deletion strain in rich microbiological medium under the no-drug condition. Interactions are color-coded, with enhancement in red (SSL) and suppression of growth defects in blue. Hierarchical clustering was performed using interaction profiles for both query deletion strains and the deletion collection strains.

Experiments were performed in biological duplicates, with four technical replicates, which correlated well (Fig. S1B), and the average standard deviation across all SGAs was 0.068. Synthetic interaction values (SIVs) for each double-deletion strain were calculated using a multiplicative approach, and the dips in the regions of the query genes due to decreased recombination efficiency were corrected as described previously (21) and in Materials and Methods. The majority of the SIVs are around 1, which indicates no interaction, while a value of <1 is an enhancing interaction and a value of >1 is a suppressing interaction. A total of ∼155,400 double-deletion strains were generated and probed in rich medium with no drug, vancomycin, and rifampin. SIVs for all generated double-deletion strains can be found in Table S2. A hierarchically clustered heat map showing all SIVs of double-deletion strains under the no-drug condition is presented in Fig. 1. Synthetic growth profiles changed when strains were exposed to antibiotic probes of membrane permeability (Fig. S2), indicating that certain double-deletion strains may be viable under the no-drug condition but are hyperpermeable to vancomycin and/or rifampin. We focused on SSL interactions, which were defined as double-deletion strains with SIVs 3 standard deviations (3σ) below the mean of the SGA, excluding outliers in the σ calculation. From our 39 SGAs, we observed approximately 30 SSL interactions on average per gene, with approximately 6 and 10 more interactions per gene in the presence of rifampin and vancomycin, respectively (Table 1). The high density of SSL interactions is shown in the center of the network maps in Fig. S3, where large nodes indicate high network connectivity. However, many outer nodes have only a single connection, showing an interaction that is specific to that gene pair (Fig. S3).

**TABLE 1:** SSL interactions across all SGAs performed^*a*^

Condition	No. of interactions	
	Total	Per gene
No drug	1,173	30.08
Rifampin	1,409	36.13
Vancomycin	1,564	40.10

a Interactions 3σ below the mean, excluding outliers, were classified as SSL interactions.

10.1128/mBio.00161-20.2 FIG S2 SGAs of outer membrane-related gene deletion strains with subinhibitory concentrations of rifampin (A) and vancomycin (B) (Fig. 1). Download FIG S2, TIF file, 2.2 MB. Copyright © 2020 Klobucar et al. 2020 Klobucar et al. This content is distributed under the terms of the Creative Commons Attribution 4.0 International license.

10.1128/mBio.00161-20.3 FIG S3 (A to C) Network interaction maps for SSL gene pairs for no drug (A), rifampin (B), and vancomycin (C). Network maps were generated in Cytoscape using an edge-weighted spring-embedded layout. Nodes were sized according to their number of edges and colored by Markov cluster (using a granularity/inflation value of 2). Self-loops due to incomplete dip correction were removed. (D) Network statistics output from Cytoscape’s NetworkAnalyzer for genetic interaction network maps in panels A to C. Download FIG S3, TIF file, 2.6 MB. Copyright © 2020 Klobucar et al. 2020 Klobucar et al. This content is distributed under the terms of the Creative Commons Attribution 4.0 International license.

10.1128/mBio.00161-20.8 TABLE S2 All synthetic interaction values for SGAs performed with no drug, vancomycin, and rifampin. Included are SSL interactions and their corresponding SIVs used to generate the network maps in Fig. S3 in the supplemental material. Download Table S2, XLSX file, 4.8 MB. Copyright © 2020 Klobucar et al. 2020 Klobucar et al. This content is distributed under the terms of the Creative Commons Attribution 4.0 International license.

### Frequent interactors in SGA analysis.

As previously noted in SGA studies (e.g., see reference 21), several recipient strains frequently formed SSL interactions with our query genes. Many of these were strains with deletions in genes that are known to be important for conjugation or recombination and are therefore unable to form double-deletion strains in SGAs. Working with only a specific subset of query genes, such as those implicated in outer membrane structure and function, makes it challenging to decipher whether the gene deletions that were frequently SSL with our query genes are simply conjugation or recombination deficient or are meaningful interactions. Gene deletion strains that appear as frequent interactors in SGAs are an important class of interactors to examine to determine the reason for the frequently detected genetic interactors and whether these should be further studied.

**FIG 2:**
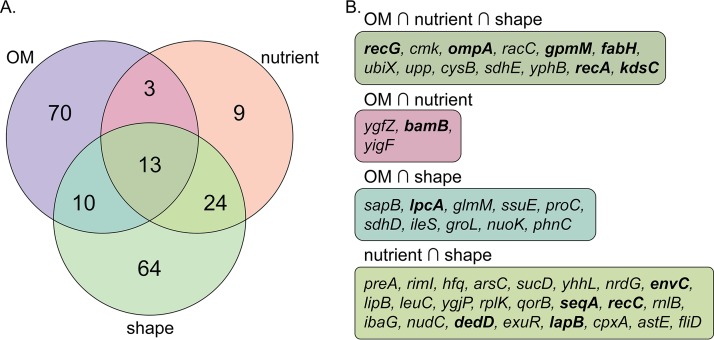
Frequent interactors in SGAs have conjugation and recombination defects. (A) Venn diagram of single-deletion strains that are frequently SSL with query strains in SGA data sets. The studies compared are the outer membrane (OM) SGAs under the no-drug condition reported here (purple), the nutrient biosynthesis SGAs reported previously (21) (orange), and the shape-related SGAs reported previously (27) (green). Single-deletion strains that were SSL (using a 2.5σ cutoff for the nutrient and shape data sets and a 3σ cutoff for the outer membrane data set) in at least 25% of crosses by the SGA data set were deemed frequent interactors. (B) Gene deletion strains that were common frequent SSL interactors between the data sets in panel A. Gene names in boldface type are those that have been previously linked to conjugation and recombination deficiencies in the literature, either directly or indirectly.

Thus, we compared the frequent SSL interactors between our outer membrane SGA data set under the no-drug condition and two previously reported SGA data sets generated in our laboratory, one of which focused on nutrient biosynthetic query gene deletions (21) and the other of which focused on query gene deletions producing shape defects (27) (Fig. 2A). A cross-gene deletion was deemed a frequent interactor if it was SSL in at least 25% of SGAs in that study. Many of the frequent interactors common between at least two of the three data sets were linked directly to having recombination or conjugation defects. For example, the Δ*recG* and Δ*recA* strains appear as frequent interactors, as the deletions in these strains are both in genes encoding double-strand break repair enzymes, which are needed for homologous recombination (36, 37). Also, the Δ*ompA* and Δ*bamB* strains appear as frequent interactors, as OmpA encodes an outer membrane protein that stabilizes mating pairs and BamB assists in inserting OmpA into the outer membrane, leading to conjugation deficiencies in strains lacking these proteins (38, 39) (Fig. 2B). Several frequent interactors were also indirectly linked to conjugation, such as the Δ*fabH* strain, as FabH is involved in fatty acid biosynthesis, which is important for membranes and cell size (40, 41), and the Δ*envC* strain, as EnvC is a peptidoglycan hydrolase activator that when deleted results in cell division and peptidoglycan defects (42).

### Profound differences in interactions when probed with Gram-positive-targeting antibiotics.

As the number of SSL interactions increased in the presence of subinhibitory concentrations of vancomycin and rifampin, this indicated that there were double-deletion strains that were more sensitive or permeable to at least one of these two antibiotics. This increased sensitivity either arose from the combined effect of both gene deletions or was a property of the single-deletion strain that was not one of our query strains since the concentrations of vancomycin and rifampin used were subinhibitory for the queries.

**FIG 3:**
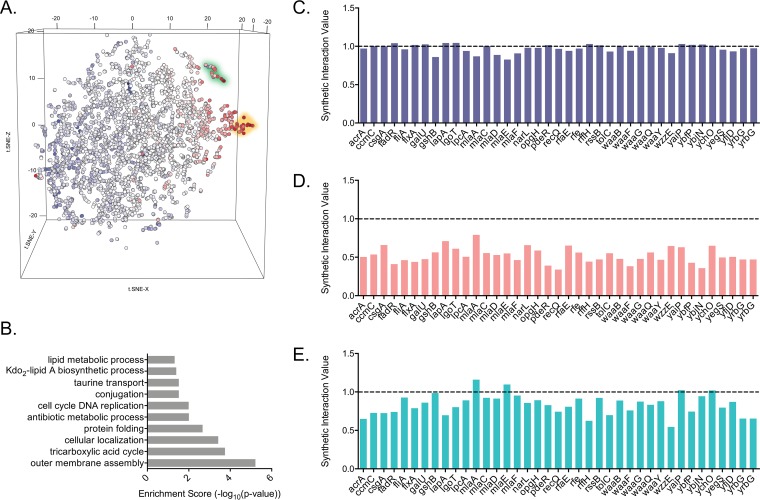
Synthetic genetic interactions are altered in the presence of subinhibitory concentrations of large-scaffold antibiotics. (A) Cluster visualization of SGAs under vancomycin stress. Dimensionality reduction using t-SNE reveals a structure of genetic interaction data specific to vancomycin stress. To highlight interactions across the vancomycin-treated array, points are colored based on the median SIV for each gene in the deletion collections. Highlighted here are two small clusters that are more SSL under vancomycin stress than under no-drug conditions. (B) GO term enrichment of the single-gene deletions from the deletion collections in the highlighted green cluster shown in panel A. The full gene list of the green cluster in panel A can be found in Table S3 in the supplemental material. (C to E) SIVs of the Δ*bepA* strain with every query gene deletion strain with no drug (C), vancomycin (D), and rifampin (E). The dotted line at 1.0 indicates no interaction.

To uncover how genetic interactions were altered in the presence of Gram-positive-targeting antibiotics, the SIVs from the vancomycin and rifampin data sets were compared to those from the no-drug data set using a *t*-distributed stochastic neighbor embedding (t-SNE) machine learning algorithm to find clusters of cross-gene deletions that differed most between data sets. When comparing the SIVs between the vancomycin and no-drug conditions, t-SNE produces clusters of cross-gene deletions that are more SSL in the presence of vancomycin (Fig. 3A; Table S3). One of these clusters is enriched for gene ontology (GO) terms notably related to outer membrane assembly, protein folding, 3-deoxy-d-manno-octulosonic acid (Kdo)_2_-lipid A biosynthesis, and lipid metabolism (Fig. 3B). One of the genes from this highlighted cluster, *bepA*, encodes the β-barrel assembly-enhancing protease. The BepA protein is known to be involved in outer membrane integrity, and deletion of the gene encoding it results in higher permeability to large-scaffold antibiotics (43). As the Δ*bepA* strain is frequently SSL with the query gene deletion strains in the presence of vancomycin and rifampin, this confirms the increased sensitivity of the strain to these antibiotics (Fig. 3C to E).

10.1128/mBio.00161-20.9 TABLE S3 Gene list from the t-SNE cluster highlighted in green in Fig. 3. Download Table S3, DOCX file, 0.01 MB. Copyright © 2020 Klobucar et al. 2020 Klobucar et al. This content is distributed under the terms of the Creative Commons Attribution 4.0 International license.

An additional cluster showing large differences between the vancomycin and the no-drug data sets is highlighted in Fig. 3A. One of the gene deletions from this cluster is the sRNA *ryjB*. Based on the frequent SSL interactions of the Δ*ryjB* strain with the query gene deletion strains, which occur exclusively under rifampin and vancomycin conditions, this implicates the sRNA in outer membrane permeability. Although this sRNA is largely uncharacterized, one group predicted the genes that it regulates using context likelihood of relatedness, an algorithm that uses transcriptional profiles to infer regulatory interactions (44). One of the inferred potential targets of RyjB was *rfaH*, a transcription antiterminator that regulates LPS production (44–46). Interestingly, the Δ*rfaH* strain also shows frequent SSL interactions under rifampin and vancomycin stress. The same clustering method was performed comparing the rifampin data set to the no-drug data set, and the bottom right cluster of red points also contains many genes involved in the outer membrane, especially LPS biogenesis, among others (Fig. S4).

10.1128/mBio.00161-20.4 FIG S4 t-SNE clustering of SGAs under rifampin stress compared to no drug. To highlight interactions across the rifampin-treated array, points are colored based on the median genetic interaction score for each gene in the deletion collections (Fig. 3). Download FIG S4, TIF file, 2.5 MB. Copyright © 2020 Klobucar et al. 2020 Klobucar et al. This content is distributed under the terms of the Creative Commons Attribution 4.0 International license.

### OMI Explorer: an online, searchable database for genome-wide interactions of outer membrane biosynthetic genes.

**FIG 4:**
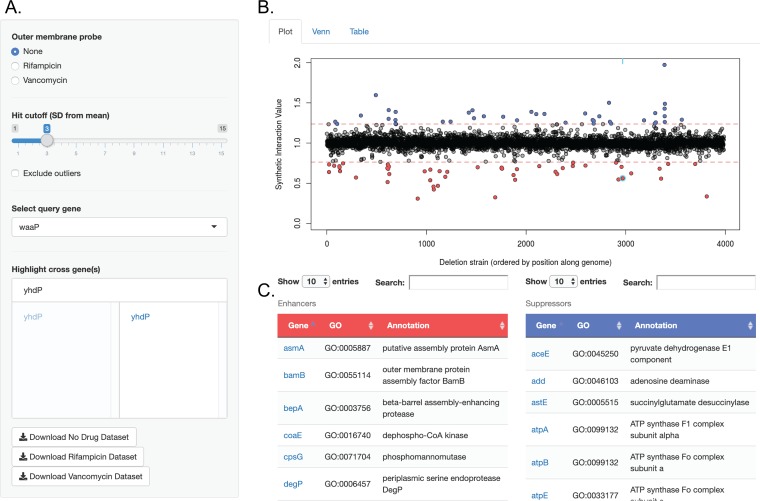
Example of a search output in the OMI Explorer. (A) The search window allows the specification of the outer membrane probe of interest, selection of the hit cutoff value based on the number of standard deviations from the mean, and selection of the query gene deletion strain of interest, and selection of which gene deletion strains in the cross are to be highlighted. (B) A plot is generated, showing the SIVs of all double-deletion strains with the specified query gene deletion. (C) GO terms and annotations for strains classified as enhancers (SSL) and suppressors based on the hit cutoff. Gene names are hyperlinked to the corresponding gene information page in the EcoCyc database.

Our accumulated data set has a total of ∼466,200 data points across all three conditions. We created an online, user-friendly database called the OMI Explorer (https://edbrownlab.shinyapps.io/omi_explorer/) to increase the accessibility of our data sets. This database allows the user to view all SGAs, which can be selected by choosing the outer membrane probe of interest (none, vancomycin, or rifampin). The user can select the desired cutoff value for which to call SSL or enhancing interactions and suppressing interactions and select the query gene of interest (Fig. 4A). In real time, a plot will be generated, displaying the SIVs of all double-deletion strains for that selected query gene along the position of the genome (Fig. 4B). Gene names of any cross-gene deletions of interest can be individually selected to appear highlighted on the plot (Fig. 4A and B). Additionally, gene names and their corresponding GO term annotations appear below the plot for significant enhancers and suppressors depending on the chosen cutoff (Fig. 4C). The gene names in these enrichment tables are hyperlinked to the genes’ corresponding EcoCyc (47) pages. Enhancing and suppressing interactions can be compared between conditions using the Venn diagram tab, and the genes in each region of intersection are displayed below. Furthermore, a table tab is included, which shows all SIVs for double-deletion strains with conditional formatting to highlight enhancers and suppressors based on the chosen standard deviation. A gene of interest can be searched within this table in order to easily view its interaction values with all query gene deletions. In all, this tool makes the data set approachable, without the need for the user to be familiar with programming.

### Interaction between Δ*yhdP* and deletions in LPS inner core biosynthesis.

**FIG 5:**
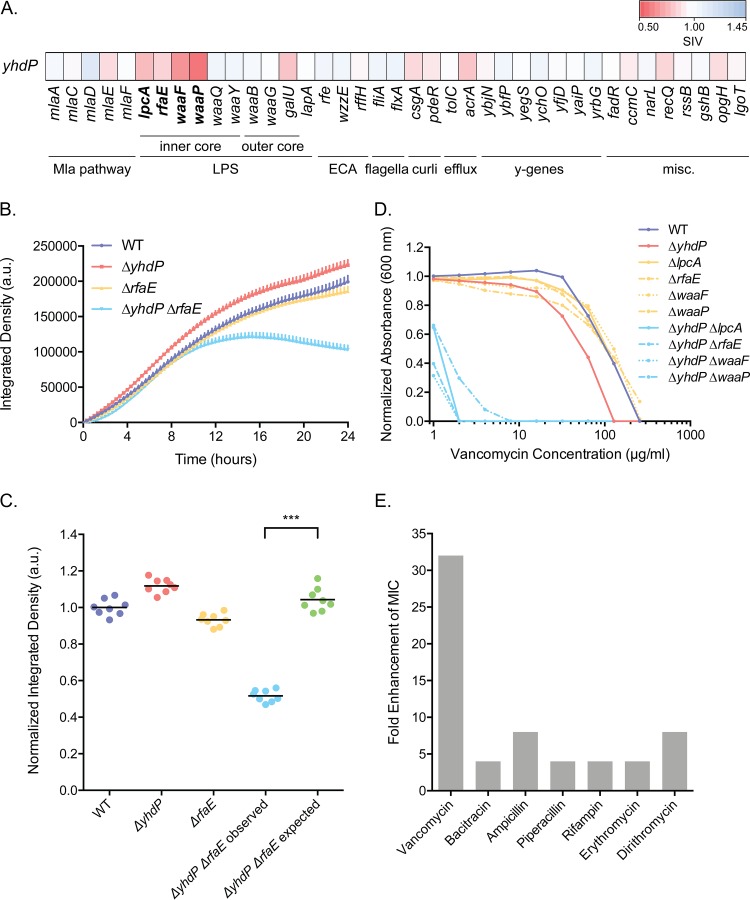
A synthetic sick interaction between Δ*yhdP* and LPS inner core deletion strains results in increased susceptibility to cell wall-active antibiotics. (A) Heat map of SIVs between the Δ*yhdP* strain and the query gene deletion strains. Red represents a lower SIV, white represents neutral interactions, and blue represents a higher SIV. (B) Growth kinetics in solid medium of the single-deletion strains compared to the double-deletion strain (*n* = 8). Error bars represent standard deviations from the means. a.u., arbitrary units. (C) Endpoint (24-h)-normalized integrated densities of the strains in panel B. Expected growth was calculated as the product of the growths of each single-deletion strain based on the multiplicative rule. Each point represents an individual replicate, and the line indicates the mean (***, *P* < 0.001 by a two-tailed Mann-Whitney test). (D) Potency analysis of vancomycin in the single- and double-deletion strains. In order to determine differences in MICs, strains were grown in the presence of 2-fold dilutions of vancomycin from 0 to 256 μg/ml, where the normalized absorbance is the background-subtracted endpoint OD_600_ divided by the background-subtracted endpoint OD_600_ in the 0-μg/ml well. The average values from three technical replicates are shown for one experiment, although experiments were performed in biological duplicate. (E) Fold enhancement (decrease) in the MICs of a subset of antibiotics in the Δ*yhdP* Δ*waaP* double-deletion strain compared to the corresponding single-deletion strains. MIC tests were performed in triplicate. All MIC values for all strains can be found in Table S4 in the supplemental material.

Using the OMI Explorer, we noticed that Δ*yhdP* showed SSL interactions with the following query deletions in LPS inner core biosynthesis: Δ*lpcA* (Δ*gmhA*), Δ*rfaE* (Δ*hldE*), Δ*waaF*, and Δ*waaP* (Fig. 5A). Deletion of *lpcA* and *rfaE* results in a heptoseless LPS consisting only of Kdo_2_-lipid A (48, 49), while deletion of *waaF* results in only one heptose on LPS (50). Deletion of *waaP* leads to a loss of core phosphates, a loss of the third heptose, as well as a lower percentage of full-length core oligosaccharide (51, 52). The function of YhdP in the cell has not yet been fully elucidated. The YhdP protein is predicted to have a transmembrane domain in the inner membrane, with the majority of its structure in the periplasmic space; it contains a domain of unknown function (DUF3971) near the middle of the protein and an AsmA2 domain at the C terminus. Recent characterization of YhdP has implicated it in the stationary-phase stress response, acting downstream of RpoS to strengthen the permeability barrier in response to sodium dodecyl sulfate (SDS) in carbon-limited media (32). Furthermore, YhdP has been linked to a role in ECA regulation (33). ECA consists of repeats of *N-*acetylglucosamine (GlcNAc), *N-*acetyl-d-mannosaminuronic acid, and 4-acetamido-4,6-dideoxy-d-galactose, and there are three different forms: cyclic ECA (ECA_cyc_) contained in the periplasm, LPS-linked ECA (ECA_LPS_), and phosphatidylglycerol-linked ECA (ECA_PG_) (reviewed in reference 53). The deletion of *yhdP* results in permeability defects, and suppression of these defects has been linked specifically to ECA_cyc_, for which the function is not well understood (33, 53). Levels of the linear ECA_LPS_ and ECA_PG_ have also been shown to be altered upon the deletion of *yhdP* (33).

10.1128/mBio.00161-20.10 TABLE S4 Antibiotic susceptibility testing of a diverse panel of antibiotics against Δ*yhdP* and LPS inner core double- and single-deletion strains. MIC values are in micrograms per milliliter. Download Table S4, XLSX file, 0.01 MB. Copyright © 2020 Klobucar et al. 2020 Klobucar et al. This content is distributed under the terms of the Creative Commons Attribution 4.0 International license.

To confirm that the Δ*yhdP* strain is synthetic sick with LPS inner core truncations, the Δ*yhdP* Δ*lpcA*, Δ*yhdP* Δ*rfaE*, Δ*yhdP* Δ*waaF*, and Δ*yhdP* Δ*waaP* double-deletion strains were remade by conjugation and PCR confirmed. Growth kinetics of the double- and single-deletion strains were monitored in solid media (31), and the double-deletion strains showed a growth defect relative to the corresponding single-deletion strains (Fig. 5B and C; Fig. S5A and B). In the Δ*yhdP* Δ*lpcA* and Δ*yhdP* Δ*rfaE* strains, the most prominent defect is in the endpoint amplitude, while in the Δ*yhdP* Δ*waaF* and Δ*yhdP* Δ*waaP* strains, along with the defect in endpoint amplitude, there also appears to be a slight defect in the growth rate (Fig. 5B and C). However, in liquid medium, the growth defect is evidenced only by a slight increase in the lag time in the double-deletion strains compared to the slowest-growing strain of the single-deletion strains (Fig. S5C).

10.1128/mBio.00161-20.5 FIG S5 Deletion of *yhdP* in LPS inner core deletion strains leads to a growth defect. (A) Growth kinetics in solid medium of the single-deletion strains compared to the double-deletion strain (*n* = 8). Error bars represent standard deviations from the mean. (B) Endpoint (24-h)-normalized integrated densities of the strains in panel A. Expected growth was calculated as the product of the growths of each single-deletion strain based on the multiplicative rule. Each point represents an individual replicate, and the line indicates the mean (***, *P* < 0.001 by a two-tailed Mann-Whitney test). (C) Growth kinetics in liquid medium of the single-deletion strains compared to the double-deletion strains (*n* = 2) (Fig. 5). Download FIG S5, TIF file, 1.3 MB. Copyright © 2020 Klobucar et al. 2020 Klobucar et al. This content is distributed under the terms of the Creative Commons Attribution 4.0 International license.

Since these Δ*yhdP* double-deletion strains with LPS inner core truncations are able to grow in liquid medium, antibiotic susceptibility testing was performed using a large panel of diverse antibiotics to determine whether the double-deletion strains were hyperpermeable (Table S4). High levels of sensitivity to the large-scaffold antibiotic vancomycin were observed in the double-deletion strains relative to the single-deletion strains (Fig. 5D and E). Increased sensitivity to bacitracin was also observed, along with a slight increase in rifampin and erythromycin sensitivity in one of the strains (Fig. 5E). This suggests a potential increase in outer membrane permeability allowing the entry of these Gram-positive-targeting antibiotics. However, enhanced MICs were also observed for other cell wall-targeting antibiotics such as ampicillin and piperacillin, suggesting that the antibiotic susceptibility of the double-deletion strains may also be due to weakened peptidoglycan (Fig. 5E).

**FIG 6:**
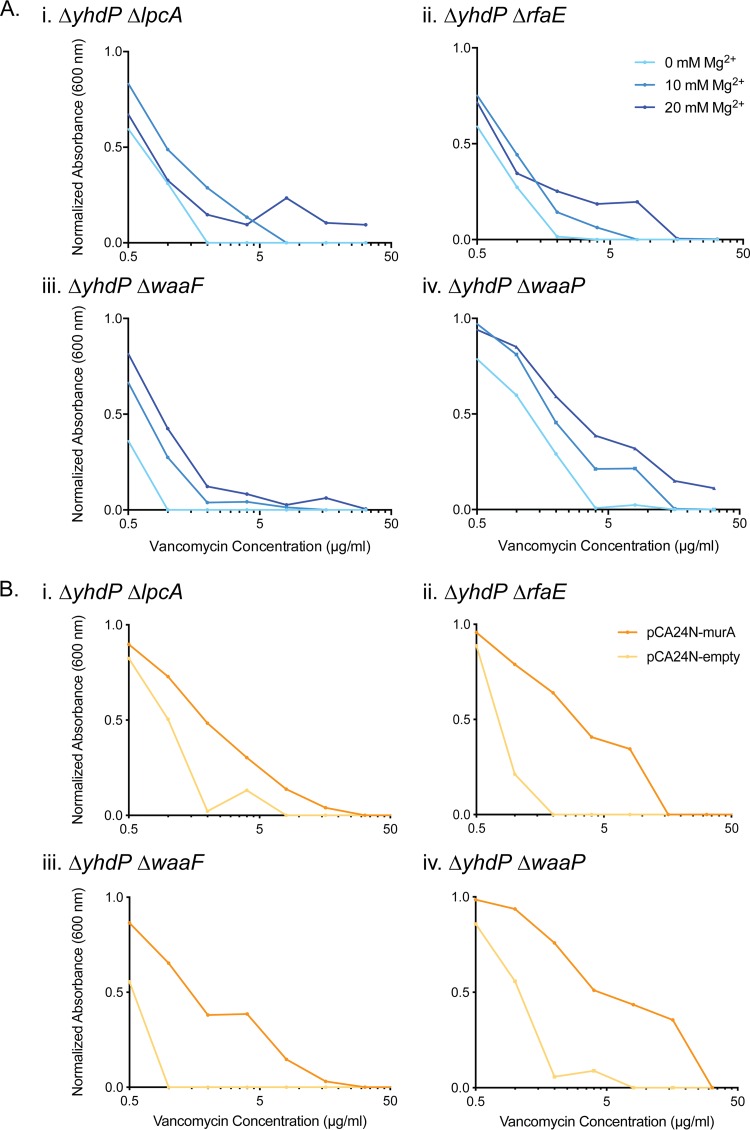
The increased susceptibility of strains with deletions in *yhdP* and LPS inner core genes to vancomycin is due to both outer membrane permeability and Und-P flux. (A) Suppression of vancomycin activity by the addition of magnesium in the Δ*yhdP* Δ*lpcA* (i), Δ*yhdP* Δ*rfaE* (ii), Δ*yhdP* Δ*waaF* (iii), and Δ*yhdP* Δ*waaP* (iv) strains. (B) Suppression of vancomycin activity by overexpression of *murA* in the Δ*yhdP* Δ*lpcA* (i), Δ*yhdP* Δ*rfaE* (ii), Δ*yhdP* Δ*waaF* (iii), and Δ*yhdP* Δ*waaP* (iv) strains. Induction using 0.1 mM isopropyl-β-d-thiogalactopyranoside (IPTG) was performed for both pCA24N-murA and pCA24N-empty (82). Strains were grown in the presence of 2-fold dilutions of vancomycin from 0 to 128 μg/ml, where the normalized absorbance is the background-subtracted endpoint OD_600_ divided by the background-subtracted endpoint OD_600_ in the 0-μg/ml well. Values shown are averages from three technical replicates. Experiments were performed in at least biological duplicate, and one representative example is shown.

To determine whether the increased susceptibility of the double-deletion strains in *yhdP* and the LPS inner core to vancomycin was due to increased outer membrane permeability, the effect of Mg^2+^ on the MIC of vancomycin was tested. Increasing levels of divalent cations such as Mg^2+^ should strengthen the outer membrane by increasing the bridging of phosphates and carboxyl functional groups between adjacent LPS molecules (15, 54). An Mg^2+^ concentration-dependent increase in the vancomycin MIC was observed in the double-deletion strains, with at least a 2- to 4-fold shift for each increase of 10 mM Mg^2+^ (Fig. 6A). Thus, outer membrane permeability is one aspect that alters vancomycin potency in these double-deletion strains.

As vancomycin targets the d-alanyl-d-alanine portion of the peptidoglycan cross-links, it is possible that the increased susceptibility of the double-deletion strains to vancomycin was also due to a weakened cell wall. The deletion of *yhdP* has previously been shown to increase the levels of ECA_LPS_ and ECA_PG_ together (33). Thus, it is possible that when LPS is altered or truncated, the deletion of *yhdP* causes an accumulation of dead-end ECA intermediates on the undecaprenyl-phosphate (Und-P) carrier, as they cannot be displayed on truncated LPS, decreasing the flux of Und-P for peptidoglycan synthesis. Indeed, this phenomenon has previously been noted for dispensable cell surface polymers, such as O-antigen and ECA, that require Und-P for synthesis (53, 55, 56). To test this, we overexpressed *murA* in the double-deletion strains. MurA catalyzes the first committed step of peptidoglycan synthesis and competes for UDP-GlcNAc with the ECA biosynthetic pathway (57). Therefore, increased levels of MurA should increase Und-P availability for peptidoglycan and decrease it for ECA (55). Indeed, the overexpression of *murA* suppressed the MIC of vancomycin by between 8- and 16-fold in each of the double-deletion strains (Fig. 6B). Thus, the susceptibility of the double-deletion strains in *yhdP* and the LPS inner core to vancomycin can also be attributed to a decreased flux of Und-P for cell wall biosynthesis as a result of ECA intermediate accumulation.

Another way to test whether the accumulation of ECA intermediates could explain, in part, the vancomycin sensitivity of double deletions between *yhdP* and LPS inner core genes was to eliminate ECA production in these strains. Rfe (WecA) transfers GlcNAc-1-phosphate onto Und-P to initiate ECA biosynthesis (58, 59). In the absence of Rfe, ECA production in the cell is abolished (55) and thus should relieve pressure on the competing peptidoglycan pathway, decreasing susceptibility to vancomycin. We constructed triple-deletion strains in which *rfe* was deleted in the Δ*yhdP* LPS mutants. The triple-deletion strains lacking ECA showed a suppression of vancomycin activity, supporting the hypothesis that in strains with a truncated or altered inner core LPS, the lack of *yhdP* leads to the sequestration of ECA by dead-end intermediates, weakening peptidoglycan (Fig. S6). However, overexpressing *murA*, disrupting ECA biosynthesis by deleting *rfe*, and increasing Mg^2+^ levels were unable to restore the growth defects observed in these double-deletion strains (data not shown). Overall, the evidence suggests that double deletions between *yhdP* and LPS inner core genes lead to increased sensitivity to cell wall-active antibiotics like vancomycin due to hyperpermeability of the outer membrane and weakened peptidoglycan due to competition with ECA for Und-P.

10.1128/mBio.00161-20.6 FIG S6 Deletion of ECA production suppresses the vancomycin MIC in the Δ*yhdP* Δ*lpcA* (A), Δ*yhdP* Δ*rfaE* (B), Δ*yhdP* Δ*waaF* (C), and Δ*yhdP* Δ*waaP* (D) strains. Triple deletions were constructed by replacing *rfe* with a chloramphenicol resistance cassette using lambda red recombineering in the double-deletion backgrounds. Values shown are averages of data from three technical replicates. Experiments were performed in at least biological duplicate, and one representative example is shown. Download FIG S6, TIF file, 0.6 MB. Copyright © 2020 Klobucar et al. 2020 Klobucar et al. This content is distributed under the terms of the Creative Commons Attribution 4.0 International license.

## DISCUSSION

Here, we probed the synthetic genetic interactions of 39 outer membrane-related genes previously implicated in permeability in E. coli (15). This was done using high-throughput bacterial conjugation to move the 39 query gene deletions into the rest of the genome-wide single-gene and sRNA deletion backgrounds (23, 30), generating double-deletion strains that were observed for SSL interactions. To increase the scope of interactions probed, the double-deletion strains were also grown in the presence of two Gram-positive-targeting antibiotics, vancomycin and rifampin, capturing double deletions that are SSL and those that result in a viable but hyperpermeable cell. Two antibiotics of different properties were chosen, as sensitivity is not necessarily generalizable across all large-scaffold antibiotics (60). This is the first E. coli genetic interaction study, to our knowledge, that probes the growth of double-deletion strains generated through SGAs on antibiotics, and it also includes the largest scale of sRNAs in the deletion strains crossed with the query strains. The percentage of SSL interactions detected in this study under the no-drug condition is on the higher end of a range similar to those reported in previous SGA studies in E. coli, where ∼0.6 to 0.8% of all double-deletion strains generated were SSL (21, 27). This suggests that the processes supporting outer membrane integrity are highly connected with each other and the rest of the cell.

In and of itself, this data set can be mined to advance knowledge gaps in the biology and interconnectivity of E. coli outer membrane pathways as well as in the permeability of the outer membrane to large-scaffold antibiotics. The data set has particular utility as a hypothesis generation tool for researchers working to further characterize outer membrane biosynthetic genes, including genes of unknown or poorly described functions. Due to the complexity and size of this data set, it has been made publicly available at https://edbrownlab.shinyapps.io/omi_explorer/ in an interactive and easily searchable format in order to encourage its use among researchers who are not versed in programming languages. Several other genetic interaction databases exist, with the most comprehensive being BioGRID (61); however, most of them do not enable visualization of the screening data for the SGAs from which they originated.

The number of SGAs in this data set provides information about frequent interactors, in addition to genetic interactions, under both no-drug and drug conditions. Determining the frequent SSL interactions under the no-drug condition reveals either genes that interact with many of the query genes in the study or those that are unable to form double-recombinant strains, due to defects in either recombination or conjugation. By comparing the frequent interactors in this data set with those from other data sets with unrelated query genes, it is possible to infer that these common frequent interactors result in one of these defects. Thus, any conjugation- or recombination-defective deletion strains discovered in low-throughput studies are likely to appear as frequent interactors here. In addition, the data set produced here reveals that there are frequent interactors in the presence of subinhibitory concentrations of vancomycin and rifampin but not under the no-drug condition. This suggests that there are underlying sensitivities of the strains with individual deletions to these antibiotics and that growth defects become evident in the SGAs where they might not be detected with traditional broth microdilution assays to determine MIC changes. For example, the Δ*malQ* strain is frequently SSL with the query genes in the presence of vancomycin but does not have a different MIC from that of the wild type (WT) (data not shown). As expected, interactions that become more intense in the presence of the Gram-positive-targeting antibiotics show enrichment for outer membrane biogenesis-related GO terms. Since the outer membrane precludes hydrophobic and large hydrophilic molecules, altering the outer membrane should increase sensitivity to these antibiotics.

In the work reported here, we reveal new chemical and genetic interactions important to our understanding of the role of YhdP, an enigmatic inner membrane protein that has been implicated in the stationary-phase stress response (32) and suggested to have a role in cyclic enterobacterial common antigen (ECA_cyc_) regulation (33). Recently, the deletion of the *yhdP* gene alone was found to result in sensitivity to SDS-EDTA and vancomycin (33). Suppressor mutants that reversed this phenotype mapped exclusively to ECA biosynthetic genes that encode the synthesis of the three forms of ECA, phosphatidylglycerol-bound ECA (ECA_PG_), LPS-associated ECA (ECA_LPS_), ECA_cyc_. Furthermore, strains lacking YhdP were shown to have decreased levels of ECA_cyc_ and increased levels of ECA_PG_ and ECA_LPS_. Selective perturbation of the three forms of ECA led to the conclusion that ECA_cyc_ in the absence of YhdP led to outer membrane damage, independent of undecaprenyl-phosphate (Und-P) pools (33).

Here, we report new interactions that shed light on the function of YhdP. Foremost were the synthetic sick interactions between the Δ*yhdP* strain and strains with deletions in LPS inner core genes in solid medium, in the absence of drugs. Furthermore, we revealed profound sensitivities of these double-deletion strains to several large-scaffold antibiotics and cell wall-targeting antibiotics, including vancomycin, which falls into both categories. This is in contrast to the comparatively subtle change in the sensitivity of the Δ*yhdP* single-gene deletion mutant to vancomycin, i.e., a 2-fold change in the MIC (33). Indeed, we have leveraged our discovery of strong synthetic sensitivity to vancomycin that results from deletions in LPS inner core genes in the Δ*yhdP* genetic background to further probe the function of the YhdP protein. In strains lacking the LPS inner core, LPS cannot be decorated with ECA (53), amplifying the significance of the regulation of ECA biosynthesis. Thus, in strains where *yhdP* is deleted and the LPS inner core is truncated, ECA_LPS_ cannot be attached to its LPS destination, which likely leads to the accumulation of dead-end ECA intermediates on Und-P, a substrate common to both ECA and peptidoglycan synthesis. Indeed, our work suggests that these dead-end intermediates reduce the availability of Und-P for peptidoglycan biosynthesis and lead to a weakened cell wall, which can be damaged more easily by cell wall-active antibiotics such as vancomycin. The vancomycin sensitivity of these double mutants is partially reversed by *murA* overexpression, to increase precursor availability for peptidoglycan biosynthesis, and by *rfe* deletion, to prevent ECA production. Vancomycin activity is also antagonized in these double-deletion strains by the addition of Mg^2+^, indicating that outer membrane permeability is an additional factor. Divalent cations are well known to strengthen outer membrane integrity (15, 54). In addition to these peptidoglycan- and permeability-related phenotypes, other factors may also influence vancomycin potency in the double-deletion strains. While cell wall defects have been linked to slower growth (62), overexpressing *murA*, disrupting ECA biosynthesis, and increasing Mg^2+^ levels were unable to restore the growth defects of the double-deletion strains in solid medium while restoring the vancomycin MIC. Nevertheless, despite previous findings showing that Und-P could not restore the permeability defect of the Δ*yhdP* strain due to ECA_cyc_ (33), Und-P becomes important in Δ*yhdP* strains in a genetic context where LPS is truncated, revealing a connection between YhdP and ECA_LPS_. This previously unknown interaction offers further insight into the enigmatic role of YhdP in E. coli.

In addition to further describing outer membrane biology in the model organism E. coli, the data described here could also provide a platform to design new antibacterial therapies for Gram-negative pathogens. SSL gene pairs could inspire chemical screens targeting these gene products. So discovered, combinations of chemicals could be used to treat pathogens in which that SSL gene pair is considered an Achilles’ heel (reviewed in reference 16). Moreover, single- or double-deletion strains that are sensitized to vancomycin and/or rifampin provide a conceivable route to enhance the activity of large-scaffold Gram-positive-only antibiotics against Gram-negative bacteria. In fact, biochemical inhibitors of some of these outer membrane biosynthetic enzymes have shown promise to this end but often lack whole-cell activity due to poor permeability or efflux (63, 64). The data set described here provides a particularly compelling discovery path that uses phenotypic screens to generate compounds with activity on whole cells. These genetic interactions have special utility as a tool for elucidating the mechanism of action of compounds thought to target one of the query deletion strains. Such compounds would not inhibit the growth of wild-type E. coli but would have a signature growth-inhibitory capacity when screened against the single-gene and sRNA deletion collections. A chemical-genetic fingerprint similar to that of one of the query genes would provide a strong hypothesis regarding the target of such a compound. Indeed, new chemical probes of a known mechanism that target outer membrane biosynthetic processes would provide great tools for further research and may have utility as leads for new therapies directed at Gram-negative pathogens (65–70).

In all, the genetic and chemical-genetic interaction networks described here provide a useful tool for the exploration of Gram-negative outer membrane biology and permeability. Furthermore, the data set provides additional opportunities for the discovery of new chemical compounds with value as probes of the biology and as leads for new drugs that target the permeability barrier of Gram-negative pathogens.

## MATERIALS AND METHODS

### Strains, gene deletions, and growth conditions.

Escherichia coli BW25113 [F^−^ Δ(*araD-araB*)*567 lacZ4787*Δ::*rrnB-3* LAM^−^ *rph-1* Δ(*rhaD-rhaB*)*568 hsdR514*] was used throughout this study to create gene deletions using an apramycin, kanamycin, or chloramphenicol resistance cassette and to perform all assays. Apramycin-resistant strains were crossed with the kanamycin-resistant Keio collection (nonessential single-gene deletions in E. coli BW25113) and the sRNA and small peptide deletion collection of E. coli MG1655 (F^−^ LAM^−^ *rph-1*) (23, 30). Bacteria were grown at 37°C for 18 to 24 h in LB (lysogeny broth) or LB agar (1.5%) with ampicillin (50 μg/ml), spectinomycin (100 μg/ml), kanamycin (50 μg/ml), apramycin (100 μg/ml), and/or chloramphenicol (25 μg/ml), if needed, unless otherwise stated. All antibiotics used in this study were purchased from Sigma-Aldrich.

The PCR products for gene deletions were generated by amplifying the apramycin resistance cassette from pSET152 linearized by EcoRI digestion (New England Biolabs). PCR amplification was performed using Phusion polymerase (Life Technologies, Inc.) and the apramycin amplification primers in Table S1 in the supplemental material, with a melting temperature of 65°C and an elongation time of 30 s. Amplification primers contain a 50-bp region of homology with the targeted region followed by 5′-AGCAAAAGGGGATGATAAGTTTATC-3′ (forward primer) and 5′-TCAGCCAATCGACTGGCGAGCGG-3′ (reverse primer).

Single-gene deletions were generated using homologous recombination (71, 72) as described previously by Côté et al. (21). Briefly, E. coli BW25113 was transformed with pSim6 (71), and cells were grown at 30°C to an optical density at 600 nm (OD_600_) of 0.8 and heat shocked at 42°C for 20 min to induce the expression of the λ phage genes *exo*, *beta*, and *gam* on pSim6. Cells were made electrocompetent, transformed with the PCR products described above, and plated on LB agar with selection. Deletions were confirmed by three PCRs using primers upstream of, downstream of, and internal to the apramycin resistance cassette (forward primer 5′-CAGAGATGATCTGCTCTGCCTG-3′ and reverse primer 5′-CAGGCAGAGCAGATCATCTCTG-3′), the kanamycin resistance cassette (forward primer 5′-CACGTACTCGGATGGAAGC-3′ and reverse primer 5′-CTTCCATCCGAGTACGTG-3′), or the chloramphenicol resistance cassette (forward primer 5′-CGATGCCATTGGGATATATC-3′ and reverse primer 5′-CAATCCCTGGGTGAGTTTCAC-3′) (Table S1).

### Synthetic genetic arrays and MIC determination on solid medium.

Synthetic genetic arrays (18, 19) were performed as described previously by Côté et al. (21). Briefly, apramycin-resistant query deletion strains were made competent for conjugation through mating with pseudo-F^+^ E. coli strains carrying a chromosomal integrative plasmid (CIP) containing the machinery required for conjugation (73). Overnight-grown cultures of the apramycin-resistant query deletion strains (in LB with apramycin) and the CIP strain (in LB with 0.3 mM diaminopimelic acid and spectinomycin) with an integration site close to the query gene were cospotted in a 1:1 ratio and incubated at 37°C overnight. Hfr strains were recovered by plating on another LB agar plate with apramycin and spectinomycin.

To determine the MICs of vancomycin and rifampin in solid LB agar medium for each apramycin-resistant Hfr query strain, cultures of the query strains in LB were arrayed in a 384-well plate in quadruplicate and pinned to a 1,536-colony density on LB agar with apramycin. Upon incubation overnight at 37°C, strains were upscaled to a 6,144-colony density on LB agar plates with various concentrations of vancomycin and rifampin (12 2-fold dilutions from 512 μg/ml to 0 μg/ml).

For the SGAs, each apramycin-resistant Hfr query strain was arrayed on LB agar with apramycin at a 1,536-colony density using the Singer Rotor HDA (Singer Instruments) and incubated overnight at 37°C. The gene and sRNA deletion collections were also arrayed at a 1,536-colony density on LB agar with kanamycin and grown at 37°C. The query strain and deletion collection colonies were copinned onto LB agar without antibiotic selection at a 1,536-colony density and incubated at 30°C for 24 h. Colonies were transferred to LB agar with apramycin and kanamycin to select for the double-deletion strains at the same colony density and incubated overnight at 37°C. Double mutants were pinned in quadruplicate at a 6,144-colony density onto LB agar, LB agar with 1/8 MIC of vancomycin, and LB agar with 1/8 MIC of rifampin (as determined in the query strain) and incubated at 37°C for 18 h. The fraction 1/8 MIC was chosen because this concentration did not inhibit growth and maximized the available amplitude to detect growth inhibition in all genetic backgrounds sensitive to the antibiotics. The deletion collections were also pinned at a 6,144-colony density in parallel to be used as controls.

### Plate imaging, quantification, and analysis.

Plates were imaged using Epson Perfection V750 scanners and analyzed as previously described (21, 31). Briefly, images were analyzed using ImageJ (74) to extract integrated density values for each colony. Edge effects were normalized using a two-pass row and column normalization system (31). An SIV was calculated for each double-deletion strain by dividing the observed normalized integrated density of the double-deletion strain by the expected integrated density of the double-deletion strain based on the product of integrated densities of the corresponding single-deletion collection strains. For the SIVs under the vancomycin and rifampin conditions, double deletions were normalized as described above for the no-drug condition, and the normalized observed integrated density of that double-deletion strain was then used as the new query strain in the triple-factor-interaction calculation. Here, the effect of the drug is represented by Δ*c*, which acts as a third deletion would in a triple mutant analysis (75), and the growth of the double-deletion strain (Δ*a*Δ*b*) under the drug condition is represented by Δ*a*Δ*b*Δ*c*: SIV (no drug) = Δ*a*Δ*b*/(Δ*a* × Δ*b*) and SIV (drug) = Δ*a*Δ*b*Δ*c*/(Δ*a*Δ*b* × Δ*c*). The dip in the index plot in the region of the query gene is corrected using a rolling median. SSL interactions are indicated by an SIV of <1, and significant interactions were identified using a 3σ cutoff.

### Data visualization.

SGA data sets were visualized in a heat map, using the heatmap.2 function from the gplots package in R. Batch effect correction was performed using the ComBat function from the sva package in R (76, 77). Genetic interaction networks were generated using the R programming language and Cytoscape (78). SSL interactions were mined using gene ontology (GO) term enrichments through EcoCyc (47) and REVIGO (79) to determine the dispensability and uniqueness of GO terms.

To visualize the overall genetic interactions within the SGAs under vancomycin and rifampin stress, we utilized t-SNE machine learning (80). This collapsed the number of dimensions to 3 while also providing a spatial structure to the data. t-SNE visualizations were prepared in OSIRIS Datawarrior (81), using a perplexity of 20, 50 source dimensions, and 1,000 iterations to structure the data.

### Growth kinetics in solid medium.

Glycerol stocks of E. coli strains in a 96-well plate were pinned using the Singer Rotor HDA in quadruplicate to a 384-colony density using 96 long pins onto an LB agar plate and incubated overnight at 37°C. The 384-source plate was pinned in duplicate using 384 short pins onto fresh LB agar plates. Plates were incubated at 37°C for 24 h and scanned every 20 min in Epson Perfection V750 scanners as described previously (31), and integrated densities were extracted for each colony. Values were background subtracted, averaged, and smoothed by locally estimated scatterplot smoothing (LOESS).

### Growth kinetics in liquid medium.

Cultures of E. coli strains were grown overnight in LB medium with appropriate antibiotic selection, if applicable. Strains were subcultured 1:50 in LB and grown at 37°C with aeration at 250 rpm to mid-log phase (OD_600_ of ∼0.4). Cells were diluted 1:10,000 in fresh LB and added to a 96-well assay plate, and the OD_600_ was monitored for 18 h at 37°C, with shaking, using a Tecan Sunrise plate reader.

### Antibiotic susceptibility testing.

Cultures of E. coli strains were grown overnight in LB medium with appropriate antibiotic selection, if applicable. Strains were subcultured 1:50 in LB medium and grown at 37°C with shaking at 250 rpm to mid-exponential phase (OD_600_ of ∼0.4). Cells were diluted 1:10,000 in fresh LB and added to a 96-well assay plate containing 2-fold dilutions of the antibiotic in either water or dimethyl sulfoxide (DMSO). Prior to incubation, the OD_600_ of assay plates was measured using the Tecan Infinite M1000 plate reader. Assay plates were incubated at 37°C with shaking (250 rpm) for 18 h, and the OD_600_ was measured. Final measurements were background subtracted and normalized to the 0-μg/ml well. The MIC was determined to be the lowest concentration that resulted in ≤10% residual growth.
