# Supplementary figures and images for: Genetic and Chemical-Genetic Interactions Map Biogenesis and Permeability Determinants of the Outer Membrane of Escherichia coli
Source: mBio. 2020 Mar 10;11(2):e00161-20. doi: 10.1128/mBio.00161-20 (PMC7064757; doi:10.1128/mBio.00161-20)

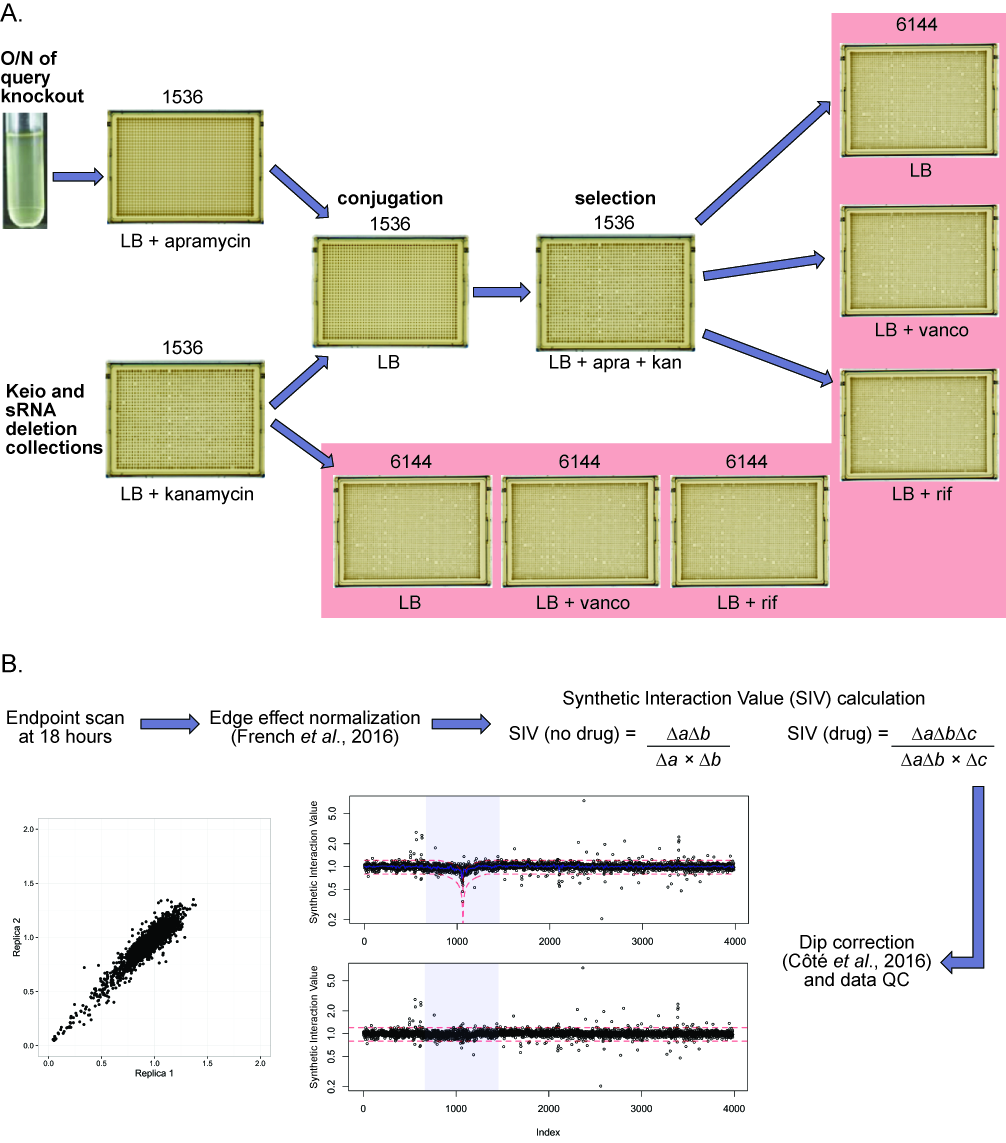

Supplement: FIG S1 [file mBio.00161-20-sf001.tif]

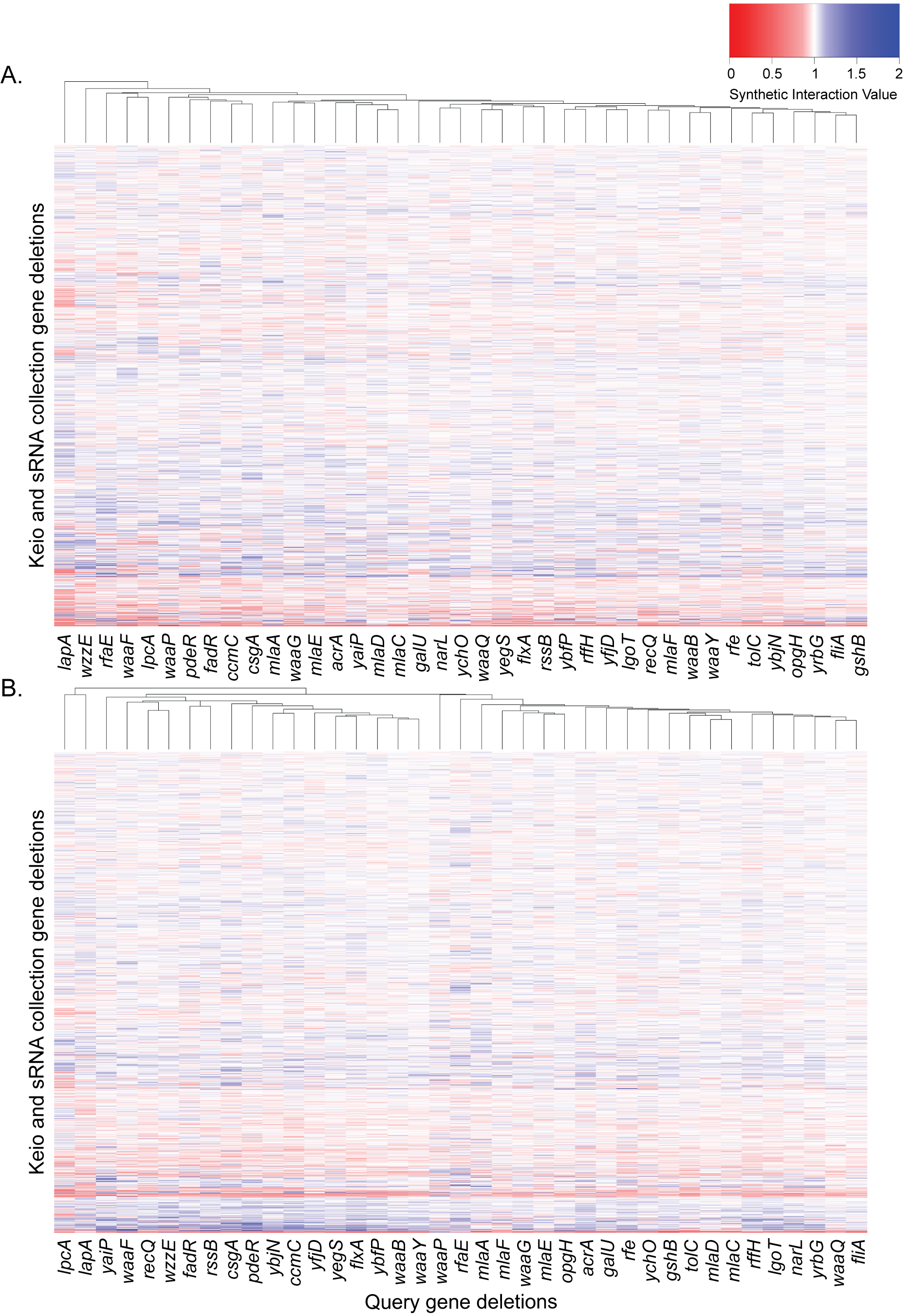

Supplement: FIG S2 [file mBio.00161-20-sf002.tif]

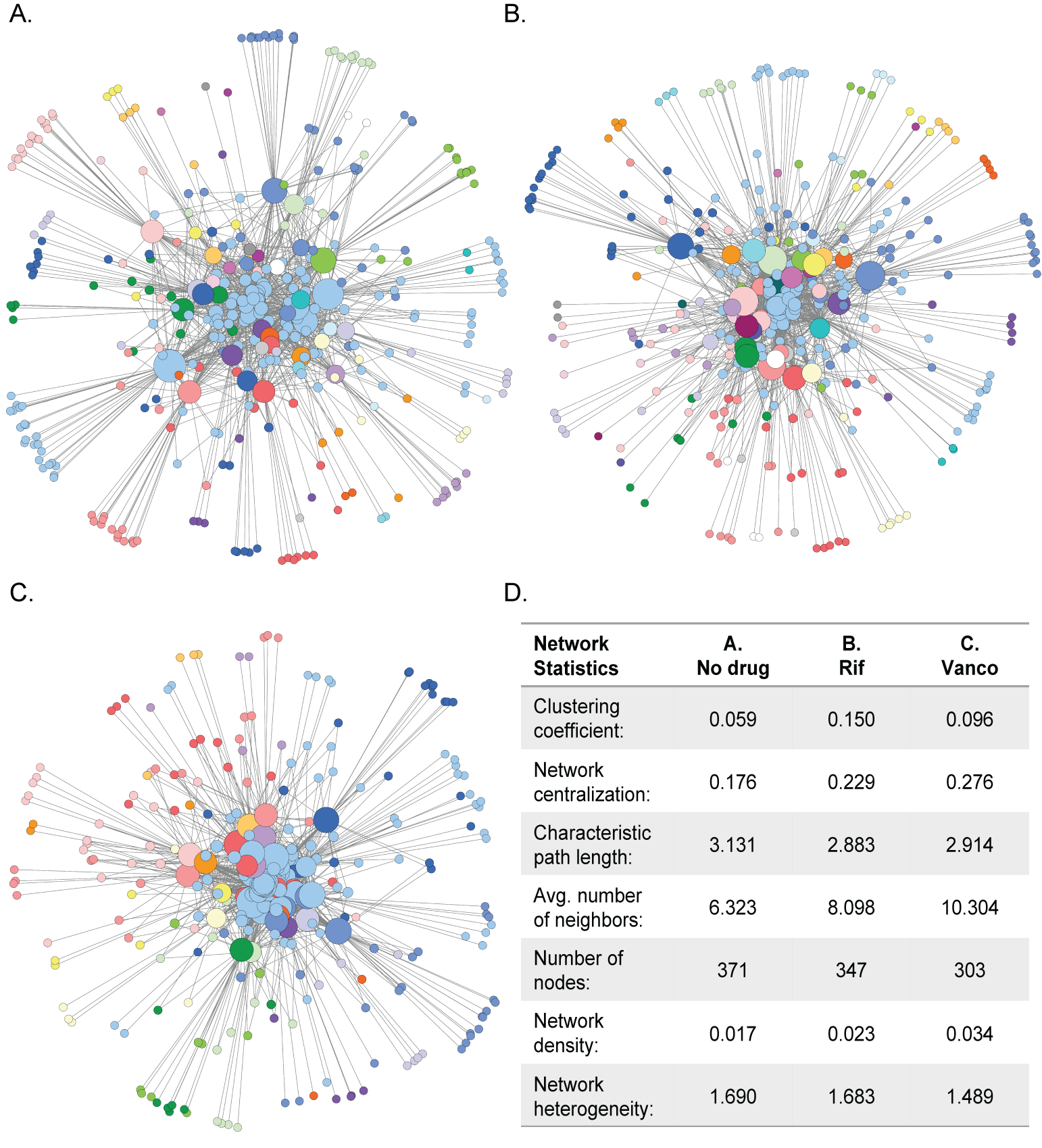

Supplement: FIG S3 [file mBio.00161-20-sf003.tif]

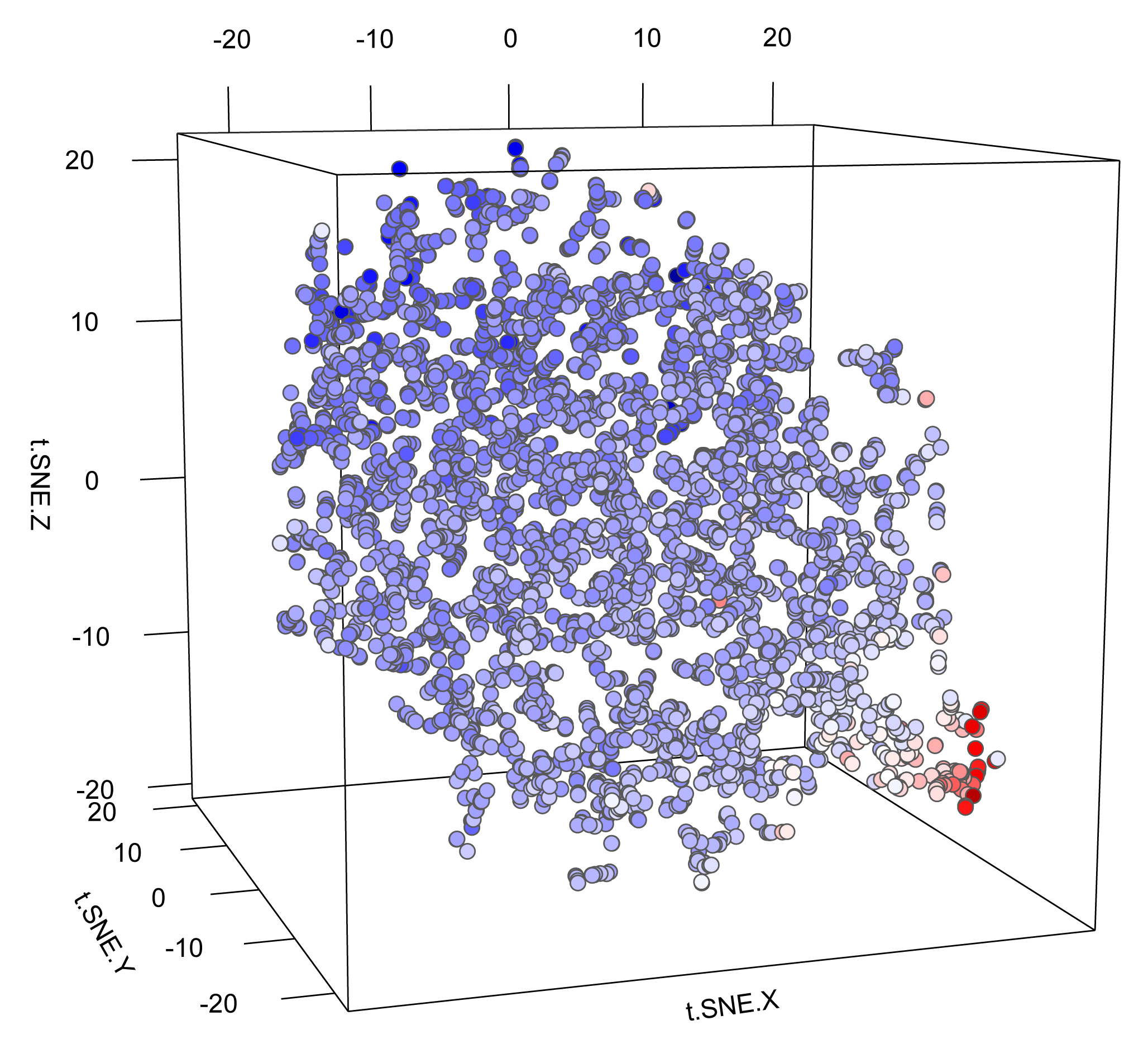

Supplement: FIG S4 [file mBio.00161-20-sf004.tif]

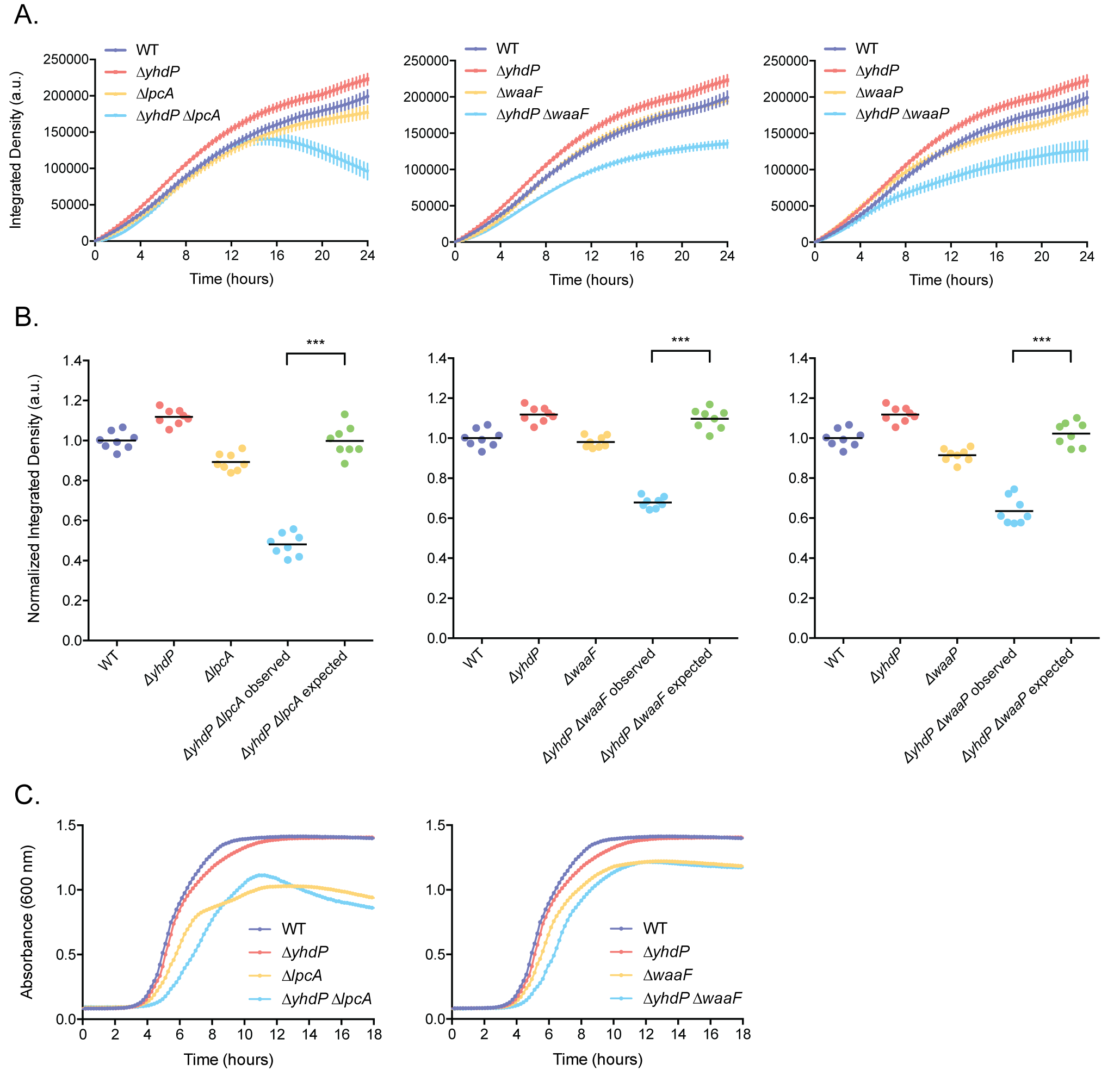

Supplement: FIG S5 [file mBio.00161-20-sf005.tif]

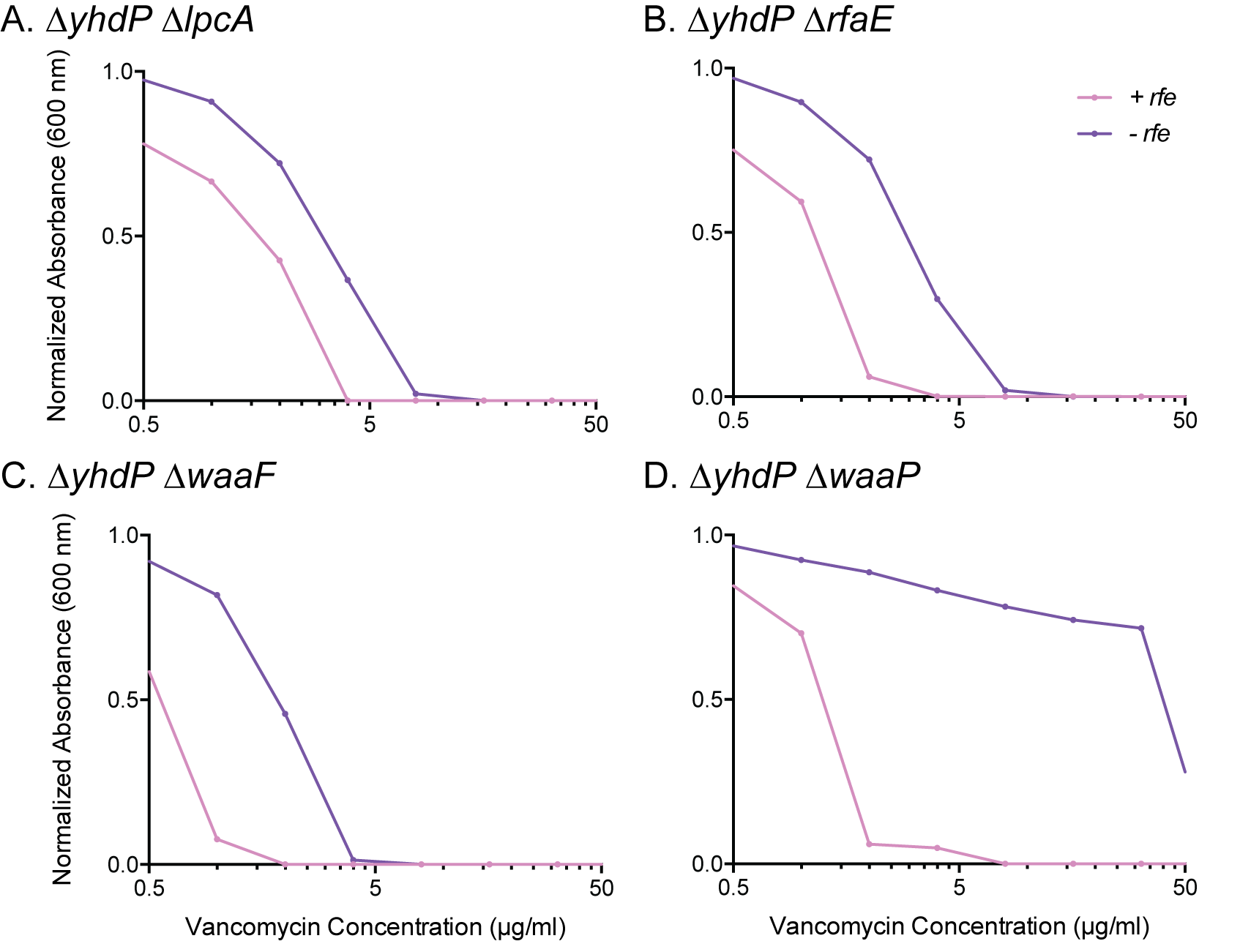

Supplement: FIG S6 [file mBio.00161-20-sf006.tif]
