# Supplementary material for: Genetic and Chemical-Genetic Interactions Map Biogenesis and Permeability Determinants of the Outer Membrane of Escherichia coli
Source: mBio. 2020 Mar 10;11(2):e00161-20. doi: 10.1128/mBio.00161-20 (PMC7064757; doi:10.1128/mBio.00161-20)
Supplement: TABLE S3 [file mBio.00161-20-st003.docx]

**Table S3: Gene list from t-SNE cluster highlighted in green in Figure 3.**

| **Gene deletion** | **Product** |
| --- | --- |
| *asmA* | putative assembly protein AsmA |
| *atl* | DNA base-flipping protein |
| *bamB* | outer membrane protein assembly factor BamB |
| *bamC* | outer membrane protein assembly factor BamC |
| *bepA* | β-barrel assembly-enhancing protease |
| *clpX* | ATP-dependent Clp protease ATP-binding subunit ClpX |
| *cydB* | cytochrome *bd*-I ubiquinol oxidase subunit II |
| *dksA* | RNA polymerase-binding transcription factor DksA |
| *fepD* | ferric enterobactin ABC transporter membrane subunit FepD |
| *gpmM* | 2,3-bisphosphoglycerate-independent phosphoglycerate mutase |
| *hns* | DNA-binding transcriptional dual regulator H-NS |
| *hupA* | DNA-binding protein HU-α |
| *icd* | isocitrate dehydrogenase |
| *ihfB* | integration host factor subunit β |
| *lapB* | lipopolysaccharide assembly protein B |
| *lpp* | murein lipoprotein |
| *lpxL* | lauroyl acyltransferase |
| *ninE* | DLP12 prophage; NinE family prophage protein |
| *nuoJ* | NADH:quinone oxidoreductase subunit J |
| *pgpB* | phosphatidylglycerophosphatase B |
| *ptsI* | PTS enzyme I |
| *rep* | ATP-dependent DNA helicase Rep |
| *rodZ* | transmembrane component of cytoskeleton |
| *rpiA* | ribose-5-phosphate isomerase A |
| *rseA* | anti-sigma-E factor RseA |
| *sdhB* | succinate:quinone oxidoreductase, iron-sulfur cluster binding protein |
| *sdhD* | succinate:quinone oxidoreductase, membrane protein SdhD |
| *secB* | SecB chaperone |
| *skp* | periplasmic chaperone Skp |
| *sucC* | succinyl-CoA synthetase subunit β |
| *tauA* | taurine ABC transporter periplasmic binding protein |
| *tolB* | Tol-Pal system periplasmic protein TolB |
| *ybcN* | DLP12 prophage; DNA base-flipping protein |
| *ybcO* | DLP12 prophage; putative nuclease YbcO |
| *ybgC* | esterase/thioesterase |
| *yedF* | putative sulfurtransferase YedF |
| *yegQ* | putative peptidase YegQ |
| *yhdP* | outer membrane permeability factor YhdP |
